# Trustworthy Cyber–Physical Edge-SHM Architecture for Operational Underground Tunnel Crack Monitoring Under Resource-Constrained Conditions

**DOI:** 10.3390/s26123958

**Published:** 2026-06-22

**Authors:** Thanh Binh Ngo, Xuan Chieu Luong, Ngoc Linh Vu, Trung Dung Bui, Quang Huy Le, Long Ngo, Quang Binh Pham, Andy Nguyen

**Affiliations:** 1 University of Transport and Communications, Hanoi 100000, Vietnam; ngobinh74@utc.edu.vn (T.B.N.); chieu1256@utc.edu.vn (X.C.L.); dungbui0132@gmail.com (T.D.B.); 2 FPT Software, Hanoi 100000, Vietnam; huylq61@fpt.com; 3 Mobifone Digital Services, Hanoi 100000, Vietnam; long.ngo@mobifone.vn; 4 Dat Phuong Group Joint Stock Company, Hanoi 100000, Vietnam; binhpq@datphuong.vn; 5 School of Science, Engineering and Digital Technologies, University of Southern Queensland, Springfield, QLD 4300, Australia

**Keywords:** structural health monitoring, crack measurement, low-cost sensors, IoT, wireless sensor networks, wavelet–Kalman filtering

## Abstract

**Highlights:**

**What are the main findings?**

- A low-cost edge-IoT-based tunnel crack monitoring architecture with lightweight secure communication was developed and validated under operational underground tunnel conditions.
- The FreeRTOS-based embedded implementation supported real-time sensing, secure LoRa/MQTT telemetry, and edge-based wavelet–Kalman processing for improved measurement stability and noise reduction.

**What are the implications of the main findings?**

- The proposed framework demonstrates the feasibility of combining low-cost edge sensing, lightweight signal processing, and secure communication for periodic underground tunnel SHM campaigns.
- Longer campaigns with more sensing nodes are needed to fully evaluate scalability, durability, and long-term reliability.

**Abstract:**

This study presents a low-cost edge-IoT-based structural health monitoring (SHM) architecture for crack monitoring in operational underground tunnels. The system integrates crack-displacement sensing, temperature measurement, ESP32-based edge processing, LoRa/MQTT communication, AES/ECDSA-based data protection, and cloud-based data management. The architecture was validated through a 30-day field campaign at two representative cracks in the Hai Van Tunnel, with measurements acquired at 60 s sampling intervals. The results show that edge-based wavelet–Kalman processing improved measurement stability and reduced high-frequency noise, while the implemented security mechanism introduced only minor latency relative to the monitoring cycle. The two monitored cracks exhibited small micrometer-scale fluctuations associated with temperature variation and showed no cumulative widening trend during the observation period. This study demonstrates the feasibility of campaign-based tunnel crack monitoring using a trustworthy edge-sensing architecture, while longer deployments with more sensing nodes are needed to fully evaluate scalability and operational durability.

## 1. Introduction

Operational SHM of underground tunnel infrastructure is important for ensuring long-term operational safety and supporting maintenance planning under complex environmental and operational conditions. Conventional tunnel inspection methods mainly rely on periodic manual surveys and visual assessment [1], which are discontinuous, labor-intensive, and often insufficient for capturing long-term structural behavior such as gradual crack evolution between inspection intervals. These limitations are particularly critical in underground tunnels, where early-stage structural deterioration may remain undetected for extended periods. Consequently, there is increasing interest in automated SHM approaches capable of providing quantitative, repeatable, and near-real-time evidence on tunnel structural conditions.

For operational tunnel management, the monitoring objective is not limited to identifying the initial cause of cracks, but also includes tracking their behaviour over repeated inspection cycles and recent validation campaigns. In practice, full permanent instrumentation of all structural components is often constrained by cost, access, power supply, and communication conditions. Campaign-based SHM therefore provides a practical pathway for repeated quantitative assessment, allowing an evolved monitoring system to be redeployed, validated, and refined as infrastructure conditions and operational requirements change over time.

Recent advances in sensing technologies, wireless sensor networks (WSNs), and Internet-of-Things (IoT) platforms have enabled distributed monitoring systems for civil infrastructure applications [2,3,4,5,6,7,8,9,10,11,12]. Smart sensing systems have been widely investigated for measuring displacement, strain, vibration, and crack-width variations using embedded sensing devices integrated with wireless communication networks. However, many existing SHM systems have primarily focused on bridges, buildings, and general smart infrastructure, where environmental conditions, accessibility, and communication constraints differ substantially from those encountered in underground tunnels.

Tunnel environments introduce several unique challenges that require dedicated SHM solutions. High humidity, dust, temperature variation, vibration disturbances, and confined geometries can degrade sensing reliability and introduce measurement noise during long-term operation. In addition, underground environments significantly affect wireless communication performance because of severe signal attenuation, multipath propagation, and constrained deployment conditions. Previous tunnel-focused studies have emphasized the importance of robust sensing architectures and field-validated monitoring systems capable of operating reliably under harsh underground conditions [13,14]. These characteristics indicate that SHM systems cannot be directly generalized across different infrastructure types and that tunnel-specific design considerations are necessary for practical long-term deployment.

Vision-based monitoring systems using CCD/CMOS cameras can provide wider spatial coverage and may also support visual tunnel inspection and traffic observation [15,16,17]. However, their suitability for micrometer-scale crack-width tracking in underground tunnels can be affected by lighting variation, dust accumulation, lens contamination, traffic occlusion, and image-quality degradation [16,17]. The present study therefore focuses on direct displacement-sensor-based monitoring for pre-identified cracks, where high measurement resolution, illumination-independent operation, low-power telemetry, and trustworthy time-series evidence are prioritized over wide-area visual coverage.

IoT-enabled SHM architectures have recently attracted considerable attention because they enable integration of sensing devices, embedded processing units, wireless communication systems, and cloud-based monitoring platforms within unified monitoring frameworks [7,18,19,20]. Such architectures support continuous data acquisition, remote accessibility, and scalable monitoring operation. Nevertheless, many existing implementations remain predominantly cloud-centric, transmitting raw sensing data to backend servers with limited edge-level processing capability. Under underground tunnel conditions, this approach may increase communication overhead, reduce scalability, and introduce unnecessary backend computational burden during long-term monitoring operation. Although edge-level signal processing has been proposed to improve sensing quality and reduce communication load, relatively few studies have experimentally evaluated lightweight edge-processing approaches under realistic tunnel deployment conditions.

Wireless communication also remains a critical challenge for underground SHM applications. Low-Power Wide-Area Network (LPWAN) technologies, particularly LoRa and LoRaWAN, have been widely investigated because of their long communication range and low energy consumption [21,22,23,24,25,26]. These characteristics make LPWAN technologies attractive for underground tunnel monitoring; however, communication performance strongly depends on physical-layer configuration, payload design, synchronization strategy, and underground propagation conditions. Many previous studies adopt communication parameters empirically without explicitly considering trade-offs among communication reliability, latency, and scalability under confined underground environments.

In addition to sensing and communication constraints, cybersecurity has emerged as an important concern for distributed IoT-based SHM systems. Compromised data integrity, unauthorized access, or malicious packet injection may lead to incorrect structural assessment and potentially unsafe maintenance decisions. Despite its importance, cybersecurity remains relatively underexplored in tunnel SHM applications, particularly for resource-constrained embedded sensing platforms [27,28]. Existing approaches frequently rely on generic protection mechanisms that are not optimized for lightweight embedded devices operating under constrained computational and communication resources. Consequently, lightweight end-to-end mechanisms capable of ensuring sensing-data authenticity, confidentiality, and trustworthiness remain insufficiently investigated for underground tunnel monitoring systems.

Tunnel-specific SHM systems reported in the literature commonly focus on crack-width monitoring, tunnel lining deformation measurement, or vibration assessment under harsh underground conditions [13]. Fiber Bragg Grating (FBG) sensing systems, wired acquisition platforms, and short-range wireless communication technologies have been investigated for tunnel monitoring applications [29,30,31]; however, many existing implementations remain limited in scalability, deployment flexibility, repeated field validation, or cybersecurity integration. In particular, short-duration experiments often provide limited evidence of operational maturity across recurring monitoring campaigns. Furthermore, relatively few studies experimentally evaluate the combined integration of lightweight edge-level signal processing, LoRa-based underground communication, and embedded cryptographic protection within operational tunnel environments.

Beyond tunnel-specific studies, recent cyber–physical SHM developments increasingly emphasize the integration of sensing, communication, edge computing, and data-security functions within unified monitoring frameworks. However, most reported implementations focus on bridges, buildings, or generic infrastructure applications, whereas tunnel environments impose additional challenges related to constrained wireless propagation, environmental variability, limited access, and long-term operational reliability. To clarify the positioning of the present work relative to recent IoT- and tunnel-oriented SHM studies, Table 1 compares the main functional elements considered in this manuscript. The comparison indicates that prior studies have made important contributions in individual areas, such as tunnel sensing, low-cost IoT monitoring, LoRa-based communication, or generic IoT security. However, the simultaneous integration of tunnel crack sensing, edge-level signal conditioning, LoRa/MQTT telemetry, lightweight cryptographic protection, and real operational tunnel deployment remains insufficiently addressed.

While previous studies have addressed individual aspects of tunnel monitoring, low-cost IoT deployment, wireless communication, edge processing, or cybersecurity, few have combined these functions within a unified tunnel-oriented SHM architecture. The proposed framework differentiates itself through the coordinated integration of embedded crack-width sensing, edge-level wavelet–Kalman signal conditioning, LoRa/MQTT telemetry, lightweight cryptographic protection, and field deployment in an operational tunnel. Accordingly, the contribution of this work lies not in introducing new standalone algorithms or communication technologies, but in validating their cyber–physical integration for trustworthy operational tunnel crack monitoring under practical underground conditions using resource-constrained ESP32-based hardware. To address these limitations, this study investigates the hypothesis that integrating lightweight edge-level signal processing and distributed cyber–physical trust mechanisms within a low-cost embedded IoT architecture can improve the reliability, stability, and trustworthiness of operational tunnel crack monitoring while maintaining compatibility with challenging underground deployment conditions.

To provide clearer operational definitions within the context of this study, the three evaluation aspects are quantified as follows:

(i) *Stability* refers to the quality and consistency of crack-width measurements after edge-level signal processing, and is quantified using signal-processing metrics including signal-to-noise ratio (SNR), root mean square error (RMSE), standard deviation, high-frequency noise energy, peak preservation ratio (PPR), and correlation (see Section 2.3).

(ii) *Reliability* refers to the system’s ability to continuously acquire, transmit, and store valid monitoring data without interruption or data loss during field deployment, as demonstrated through sustained field operation, communication continuity, and consistent data availability (see Section 2.4, Section 3 and Section 3.4).

(iii) *Trustworthiness* refers to the integrity, authenticity, and security of monitoring data, and is quantified through cybersecurity validation metrics, including attack detection rate, verification latency, and successful cryptographic authentication using AES/ECDSA mechanisms (see Section 2.5, Section 2.6.5 and Section 3.4).

Based on this motivation, a low-cost IoT-based SHM framework is proposed for operational crack monitoring in underground tunnel infrastructure. The proposed architecture integrates embedded crack-width sensing, ESP32-based edge signal conditioning, LoRa/MQTT communication, lightweight cryptographic authentication, and cloud-based time-series data management within a unified monitoring framework designed specifically for repeated underground tunnel deployment. A combined wavelet–Kalman filtering approach is implemented on embedded edge devices to improve crack measurement stability and suppress environmental noise under harsh underground conditions. In addition, lightweight elliptic-curve-based authentication and AES-encrypted communication mechanisms are incorporated to improve sensing data trustworthiness during distributed monitoring operation.

The contribution of this study therefore lies in the system-level validation of a trustworthy edge-SHM workflow for operational tunnel crack monitoring. Accordingly, the main contributions of this study are as follows:

- Development of a low-cost embedded IoT-based SHM framework for operational underground tunnel crack monitoring;
- Integration and quantitative evaluation of lightweight wavelet–Kalman edge signal processing for improving crack measurement stability under underground operational conditions;
- Implementation of lightweight ECC-based authentication and encrypted communication mechanisms for secure distributed sensing in tunnel SHM applications;
- Experimental deployment and validation of the proposed framework in the Hai Van tunnel infrastructure under realistic monitoring conditions.

## 2. Methodology

### 2.1. Overall Hai Van Tunnel and SHM–IoT Framework

The Hai Van Tunnel operates under heavy traffic, humid underground conditions, temperature variation, and disturbances associated with nearby construction. These conditions motivate repeatable quantitative crack monitoring rather than relying only on periodic visual inspection.

**Figure 1:**
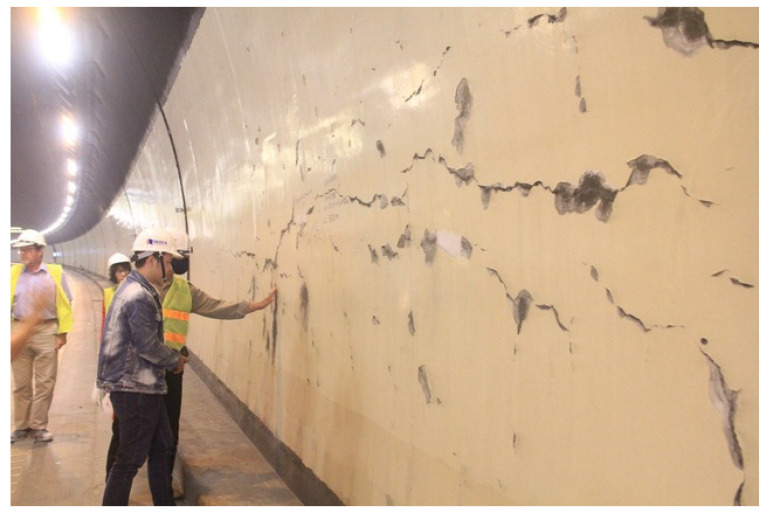
Hai Van Tunnel environment and lining structure where crack monitoring sensors were deployed.

Figure 1 shows the Hai Van Tunnel environment used for the operational crack-monitoring campaign.

Localized cracking and limited inspection accessibility make the Hai Van Tunnel a representative environment for operational SHM validation. The proposed framework therefore focuses on crack-width acquisition, environmental co-measurement, edge stabilization, wireless transmission, and cloud-based evidence management.

The system architecture is organized into four operational layers: tunnel-mounted crack and temperature sensing, ESP32-based edge acquisition and preprocessing, LoRa/MQTT communication, and cloud storage with dashboard-assisted evidence review.

**Figure 2:**
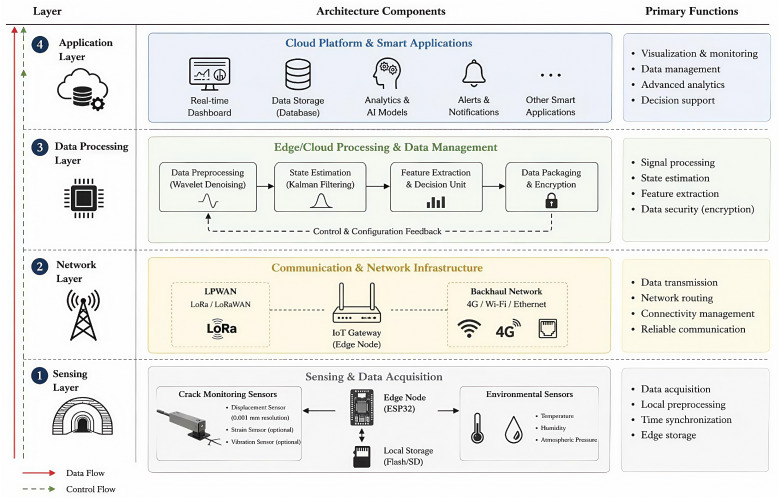
Operational architecture of the proposed IoT-based SHM framework, linking field sensing, edge processing, secure telemetry, and cloud-based evidence management.

Figure 2 presents the overall operational architecture of the proposed edge-IoT SHM framework.

Wavelet denoising [32] and Kalman filtering [33] are executed at the edge to stabilize measurements before secure transmission. The gateway forwards authenticated records to the cloud platform, where data are stored, displayed, and exported for offline structural interpretation.

### 2.2. Sensing and Edge-Level Signal Processing

Crack-width measurements in tunnels are affected by humidity, temperature variation, vibration, electromagnetic interference, and sensor drift. Each ESP32 sensing node therefore performs wavelet denoising and recursive Kalman filtering before communication. The output is an edge-conditioned sensing measurement; higher-level structural interpretation is deferred to offline analysis.

**Figure 3:**
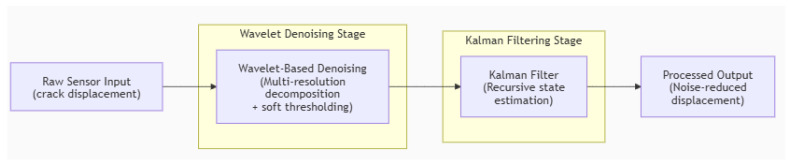
Overview of the edge-level signal conditioning pipeline.

Figure 3 summarizes the edge-level signal conditioning pipeline applied before secure data transmission.

The edge-processing pipeline transforms raw ADC readings into edge-conditioned measurements before secure transmission, reducing communication of noisy data and supporting more stable downstream interpretation.

The raw crack-width measurement at discrete time step *k* is modeled as follows:

(1) $$D_{\mathrm{raw}}\left(k\right)=S_{\mathrm{true}}\left(k\right)+\varepsilon_{\mathrm{noise}}\left(k\right)+\varepsilon_{\mathrm{drift}}\left(k\right)$$

where $S_{\mathrm{true}}\left(k\right)$ denotes the true structural crack displacement, $\varepsilon_{\mathrm{noise}}\left(k\right)$ represents high-frequency stochastic measurement noise, and $\varepsilon_{\mathrm{drift}}\left(k\right)$ captures slow-varying environmental and sensor drift components.

**Wavelet-Based Denoising
**

Wavelet denoising suppresses high-frequency disturbances while preserving the low-frequency crack-width trend. The implementation uses Daubechies-4 (db4), four decomposition levels, Donoho universal thresholding (VisuShrink), and soft-threshold reconstruction.

The selection of the wavelet parameters in this study follows established SHM signal-processing practice and practical constraints associated with embedded implementation. The Daubechies-4 (db4) wavelet was adopted due to its compact support, favorable time–frequency localization properties, and extensive use in vibration and displacement denoising for structural-health-monitoring applications [32,34,35]. These characteristics enable effective separation of measurement noise from slowly varying structural responses.

The use of four decomposition levels was selected to balance the separation of high-frequency tunnel-induced disturbances, such as traffic vibration and environmental noise, from the low-frequency crack-evolution trend while maintaining computational efficiency for real-time execution on ESP32 platforms. Increasing the number of decomposition levels would provide finer frequency separation but would also increase computational cost without substantial practical benefit for the present monitoring scenario.

VisuShrink with soft thresholding was employed due to its simplicity, robustness, and low computational overhead compared with more complex adaptive thresholding methods [36]. This choice is particularly suitable for resource-constrained edge devices where deterministic execution time and implementation reliability are critical.

It is noted that the focus of this study is on system-level deployment and validation rather than optimization of signal-processing parameters. Therefore, a comprehensive sensitivity analysis of wavelet configurations is beyond the scope of the present work. The selected parameters represent a practical and literature-consistent configuration suitable for real-time tunnel monitoring under resource-constrained conditions.

The noise standard deviation is estimated using the median absolute deviation of the first-level detail coefficients:

(2) $$\sigma =\frac{\mathrm{median}(|d_{1}|)}{0.6745}$$

The threshold is computed using:

(3) $$\lambda =\sigma \sqrt{2\log N}$$

where *N* denotes the signal length.

**Kalman Filtering for Recursive State Estimation**

Following wavelet denoising, a recursive Kalman filter estimates the stabilized crack displacement trajectory.

The state vector is defined as follows:

(4) $$x_{k}=\left[\begin{array}{c} w_{k}\\ {\dot{w}}_{k} \end{array}\right]$$

Assuming a constant-velocity model:

(5) $$x_{k}=Ax_{k-1}+w_{k}$$

with

(6) $$A=\left[\begin{array}{cc} 1 & \Delta t\\ 0 & 1 \end{array}\right]$$

The process-noise covariance is modeled as follows:

(7) $$Q=q\left[\begin{array}{cc} \frac{\Delta t^{4}}{4} & \frac{\Delta t^{3}}{2}\\ \frac{\Delta t^{3}}{2} & \Delta t^{2} \end{array}\right]$$

where $q=10^{-6}$ was empirically tuned to balance responsiveness and noise suppression under realistic tunnel conditions.

The observation model is:

(8) $$z_{k}=Hx_{k}+v_{k}$$

with

(9) $$H=\left[\begin{array}{cc} 1 & 0 \end{array}\right]$$

and $v_{k}∼N\left(0,R\right)$, where $R=10^{-6}\;{\mathrm{mm}}^{2}$ is estimated based on sensor resolution and residual noise.

The final processed output is:

(10) $$D_{\mathrm{proc}}\left(k\right)={\hat{w}}_{k}$$

**Temperature Compensation**

Thermally induced variations are compensated using:

(11) $$w_{c}=w_{m}-\alpha \left(T-T_{\mathrm{ref}}\right)$$

where α is the experimentally estimated thermal coefficient.

The thermal coefficient α was experimentally determined through controlled laboratory calibration by correlating sensor output variation with temperature changes under fixed displacement conditions. This calibration procedure ensures that the coefficient reflects the inherent thermo-mechanical response of the sensing system rather than structural crack behavior.

In the context of this study, the temperature compensation model is introduced primarily to illustrate the integration of thermo-mechanical effects within the proposed SHM–IoT framework, rather than to establish a generalized predictive model of temperature-dependent crack behavior. During the 30-day field campaign, the monitored cracks exhibited only small and largely reversible fluctuations, which limited the applicability of quantitative validation of the compensation model.

Accordingly, the field measurements were used mainly for qualitative interpretation of thermo-mechanical response, while a more comprehensive evaluation of temperature compensation performance is identified as a direction for future work.

**Edge–Cloud Data-Handling Principle**

Edge processing is used for signal stabilization and physical plausibility checking before transmission. Validated raw measurements and corresponding edge-conditioned values are retained for traceability and offline re-analysis.

The overall processing can be summarized as follows:

(12) $$D_{\mathrm{proc}}=\mathrm{Kalman}\left(\mathrm{Wavelet}(D_{\mathrm{raw}})\right)$$

Filtering is executed on the ESP32 using 32-bit floating-point arithmetic with per-sample recursive updates.

### 2.3. Effectiveness of Signal Processing on Crack Width Measurements

The signal-processing evaluation focuses on suppressing tunnel-induced noise while preserving crack-evolution trends.

The evaluated metrics include SNR, RMSE, standard deviation, high-frequency noise energy, peak preservation ratio (PPR), and correlation.

The signal-to-noise ratio is defined as follows:

(13) $$\mathrm{SNR}=10\log_{10}\left(\frac{P_{\mathrm{signal}}}{P_{\mathrm{noise}}}\right)$$

The root mean square error (RMSE) is computed as follows:

(14) $$\mathrm{RMSE}=\sqrt{\frac{1}{N}\sum_{i=1}^{N}{\left(x_{i}-{\hat{x}}_{i}\right)}^{2}}$$

The correlation coefficient is given by:

(15) $$r=\frac{\sum_{i=1}^{N}\left(x_{i}-\bar{x}\right)\left(y_{i}-\bar{y}\right)}{\sqrt{\sum_{i=1}^{N}{\left(x_{i}-\bar{x}\right)}^{2}\sum_{i=1}^{N}{\left(y_{i}-\bar{y}\right)}^{2}}}$$

The peak preservation ratio (PPR) is defined as follows:

(16) $$\mathrm{PPR}=\frac{A_{\mathrm{filtered}}}{A_{\mathrm{raw}}}$$

**Dataset Description**

The dataset consists of S2 measurements at Km07 + 231 collected over 24 h at a 60 s interval.

**Qualitative Evaluation of Filtering Performance**

The qualitative filtering result shows that high-frequency oscillations were reduced, while the low-frequency crack-width trend was preserved, which is essential for avoiding overinterpretation of sensor noise as structural response.

**Figure 4:**
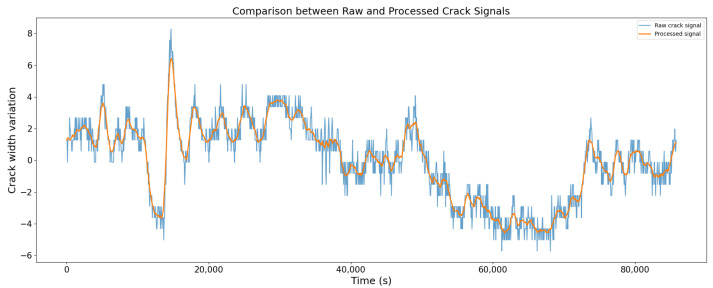
Raw and edge-conditioned S2 crack-width measurements, demonstrating noise reduction while retaining the low-frequency structural trend.

Figure 4 compares the raw and edge-conditioned S2 crack-width measurements.

**Local Noise-Suppression Analysis**

At the local scale, the processed signal suppresses short-period disturbances without introducing obvious temporal distortion.

**Figure 5:**
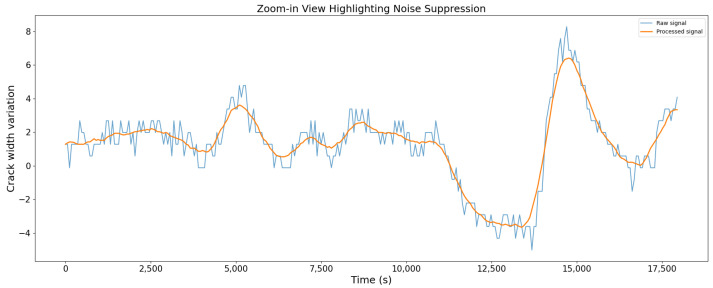
Local comparison showing suppression of high-frequency disturbances without visible temporal distortion.

Figure 5 provides a local view of the noise-suppression effect.

**Global Trend Preservation**

At the campaign scale, the processed trajectory retains the global crack-width trend required for structural interpretation.

**Figure 6:**
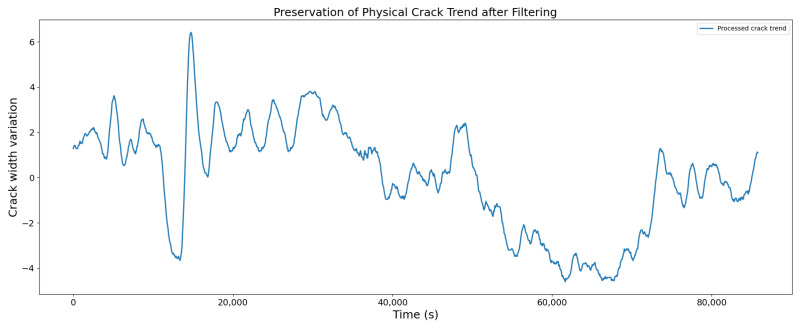
Processed crack-width trajectory used to verify preservation of campaign-scale trend information.

Figure 6 verifies that the campaign-scale trend is preserved after edge conditioning.

**Quantitative Evaluation**

The quantitative metrics confirm that the combined wavelet–Kalman framework improves signal quality while preserving relevant crack-response features.

Table 2 summarizes the quantitative signal-processing performance metrics used to evaluate noise reduction and trend preservation.

The combined wavelet–Kalman pipeline improves noise attenuation and temporal consistency compared with raw measurements.

Although the quantitative evaluation is presented using the representative 24-h S2 dataset, qualitatively similar filtering behavior was observed at S1 and throughout the remainder of the monitoring campaign when the same processing pipeline was applied.

**Discussion and Insight**

Key observations are:

- Wavelet denoising effectively attenuates high-frequency disturbances associated with vibration, electromagnetic interference, and ADC quantization noise.
- Recursive Kalman filtering improves temporal consistency through dynamic state estimation.
- The combined approach preserves both local crack-response characteristics and long-term structural trends critical for reliable SHM interpretation.

Overall, the edge-processing strategy improves crack-width stability under challenging tunnel monitoring conditions.

### 2.4. Communication Architecture

The communication architecture follows a deterministic node–gateway–cloud hierarchy using LoRa, TDMA scheduling, and MQTT forwarding. LoRa Time-on-Air (ToA) is used to size TDMA slots and estimate latency/scalability.

#### 2.4.1. LoRa Transmission Model and Physical-Layer Analysis

LoRa communication is configured using the spreading factor (SF), bandwidth (BW), coding rate (CR), and payload length. These parameters jointly determine symbol duration and packet transmission time. The symbol duration is defined as follows:

(17) $$T_{s}=\frac{2^{SF}}{BW}.$$

Increasing the spreading factor improves communication robustness, which is particularly important in tunnel environments with severe signal attenuation. However, this improvement comes at the cost of longer symbol duration and increased transmission latency.

Each LoRa packet consists of a preamble and a payload. The preamble duration is given by:

(18) $$T_{\mathrm{pre}}=\left(N_{\mathrm{pre}}+4.25\right)T_{s},$$

where the constant 4.25 accounts for synchronization overhead defined by the LoRa PHY specification.

The number of payload symbols depends on the payload size and PHY configuration:

(19) $$N_{\mathrm{pl}}=8+\max\left(\left[\frac{8PL-4SF+28+16CRC-20IH}{4(SF-2DE)}\right]\left(CR+4\right),0\right).$$

The corresponding payload transmission time is:

(20) $$T_{\mathrm{pl}}=N_{\mathrm{pl}}\;T_{s},$$

leading to the total packet Time-on-Air:

(21) $$T_{\mathrm{ToA}}=T_{\mathrm{pre}}+T_{\mathrm{pl}}.$$

Time-on-Air represents the minimum airtime required for a single uplink transmission and forms the fundamental constraint for MAC-layer scheduling in LoRa-based tunnel monitoring networks.

#### 2.4.2. TDMA Slot Design and Network Scalability

To avoid packet collisions and ensure deterministic behavior when multiple sensor nodes operate concurrently, a  Time Division Multiple Access (TDMA) scheme is implemented on top of the LoRa physical layer.

**Figure 7:**
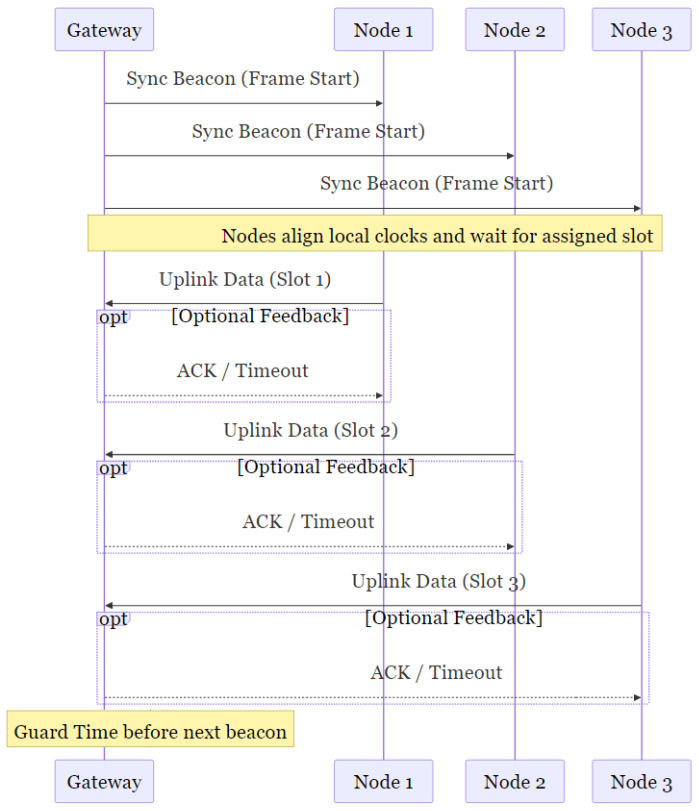
Event-driven TDMA communication between gateway and sensor nodes initiated by synchronization beacons.

Figure 7 illustrates the event-driven TDMA communication process between the gateway and sensor nodes.

The TDMA procedure reduces collision risk by allowing the gateway to broadcast a synchronization beacon at the start of each frame; sensor nodes then transmit only in assigned slots sized from the LoRa ToA analysis plus guard time.

**Figure 8:**
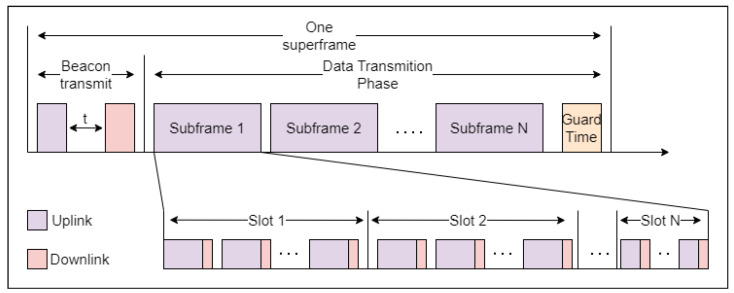
TDMA superframe structure with beacon synchronization and time-slotted uplink/ downlink transmission.

Figure 8 shows the TDMA superframe structure used for beacon synchronization and scheduled uplink/downlink transmission.

The beacon-synchronized superframe allocates deterministic uplink and downlink opportunities for each node. In practical operation, additional timing overheads must be considered, including RF transceiver switching delay and clock drift. Accordingly, the TDMA slot duration is defined as follows:

(22) $$T_{\mathrm{slot}}=T_{\mathrm{ToA}}+T_{\mathrm{RF}}+T_{\mathrm{guard}}.$$

Given a frame duration $T_{\mathrm{frame}}$, the maximum number of sensor nodes supported within one TDMA frame is:

(23) $$N_{\max}=\left\lfloor \frac{T_{\mathrm{frame}}}{T_{\mathrm{slot}}}\right\rfloor .$$

The resulting LoRa ToA and TDMA slot lengths for different spreading factors are summarized in Table 3.

The results illustrate the fundamental trade-off between communication robustness and network scalability: higher spreading factors improve link reliability but significantly reduce the number of nodes that can be scheduled within a TDMA frame. In practical tunnel deployments, the spreading factor should be selected according to the required communication range, tunnel geometry, and expected network size. Lower spreading factors (e.g., SF7–SF8) are generally preferable when link quality is sufficient because they reduce packet airtime and increase network capacity. Higher spreading factors (e.g., SF10–SF12) may be required in longer tunnel sections or challenging propagation environments, although this reduces the maximum number of supported nodes. Therefore, SF selection should balance communication reliability and network scalability according to site-specific deployment requirements.

#### 2.4.3. MQTT-Based Gateway-to-Cloud Communication

After LoRa uplink reception, the gateway forwards packets to the cloud backend using MQTT. MQTT provides a lightweight publish–subscribe interface between low-power sensing and cloud storage. The gateway only republishes encrypted packets; payload decryption, verification, and analysis are performed at the backend.

The MQTT topic structure follows a hierarchical naming scheme:

(24) tunnel/{node_id}/measurement

where {node_id} identifies the physical monitoring location (e.g., S1, S2). This structure supports scalable multi-node tunnel deployments and efficient data subscription.

The gateway–cloud message trace provides implementation evidence that authenticated monitoring records can be forwarded through the MQTT interface during field operation.

Upon backend reception, packets are verified, decrypted, parsed, and stored by sensor identifier and timestamp. Derived quantities, such as displacement variation, are computed offline using:

(25) Δ=adc−mean,

where the reference mean may be defined globally or through a sliding window.

Details of the payload structure, encryption, and authentication mechanisms are presented in the following Section 2.5.

**Figure 9:**
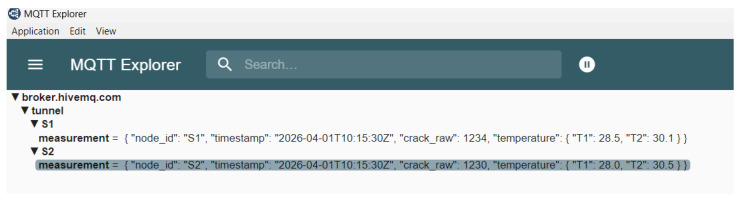
Gateway–cloud MQTT trace demonstrating field-data forwarding from tunnel sensor nodes to the backend.

Figure 9 provides an example gateway–cloud MQTT trace from the field-data forwarding process.

### 2.5. Security Architecture for SHM–IoT System

#### 2.5.1. Security Architecture Overview

Reliable communication is essential for operational SHM in underground infrastructure systems. The proposed SHM system adopts an IoT communication framework based on the Message Queuing Telemetry Transport (MQTT) protocol, which is widely used in distributed sensing applications due to its lightweight architecture and low bandwidth requirements.

In the proposed framework, each ESP32 sensor node acquires crack and environmental signals, applies wavelet denoising and Kalman filtering, serializes the resulting edge-conditioned sensing measurements, encrypts the payload using AES, and signs the ciphertext using ECDSA. The LoRa gateway forwards the encrypted and signed packets to the MQTT broker and cloud monitoring platform without plaintext access. This publish–subscribe architecture allows for efficient data exchange and supports scalable integration of multiple sensor nodes deployed throughout the tunnel.

The workflow is: ADC acquisition, edge conditioning, compact payload construction, AES encryption, ECDSA signing, LoRa transmission, MQTT forwarding, backend verification/decryption, and database insertion.

#### 2.5.2. Multi-Layer Data-Centric Security Architecture and Cryptographic Mechanisms

The proposed SHM–IoT system uses data-centric security across the edge, communication, and cloud layers to protect confidentiality, integrity, and authenticity of monitoring records.

**Figure 10:**
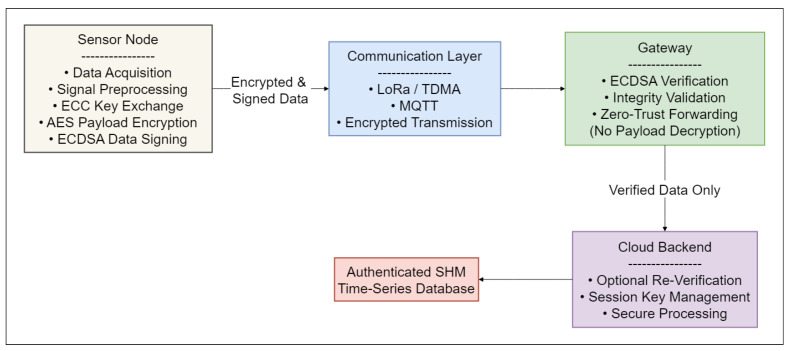
High-level overview of the data-centric security architecture.

Figure 10 summarizes the multi-layer security architecture used in the proposed SHM–IoT system.

In this architecture, measurements are encrypted and signed at the sensor node, forwarded over LoRa and MQTT without gateway plaintext access, and accepted into the cloud database only after backend verification and decryption.

#### 2.5.3. Cryptographic Design Rationale

ECC is adopted because it provides public-key authentication with smaller keys than RSA on resource-constrained ESP32 nodes. ECDH establishes session keys, ECDSA authenticates payloads, and AES protects monitoring records after session establishment.

#### 2.5.4. Secure Data Processing and Transmission Workflow

After edge signal conditioning, each monitoring record is protected using the following encrypt–then–sign workflow:

ADC Acquisition → Wavelet Denoising → Kalman Filtering → Conditioned Measurements → Binary Payload Construction → AES Encryption → ECDSA Signature → LoRa Transmission.

**Cryptographic configuration.
**

- No, and the present should be kept

The cryptographic implementation is designed to balance security and computational efficiency on resource-constrained ESP32 platforms. The following configurations are used:

- AES-128 encryption: payloads are encrypted using AES-128 in Electronic Codebook (ECB) mode.
- Hashing: SHA-256 is used to generate message digests for integrity protection.
- Digital signature: ECDSA is implemented on the secp256r1 (NIST P-256) elliptic curve.
- Key establishment: session keys are derived using Elliptic Curve Diffie–Hellman (ECDH).
- Entropy source: a hardware true random number generator (TRNG) on the ESP32 is used for cryptographic operations.

**Backend public key management.**

The backend identifies each sensor node using a pre-registered public key mapped to its node identifier. These keys are provisioned during system setup and securely stored in the backend database.

The gateway does not participate in key management and does not have access to cryptographic keys or plaintext payloads, ensuring a zero-trust architecture.

**Encryption and signing process.**

At the sensor node, the plaintext sensing payload *P* is first encrypted using symmetric AES encryption with a session key $K_{s}$:

(26) $$C=AES_{K_{s}}\left(P\right)$$

where *C* denotes the resulting ciphertext. To ensure data integrity and source authenticity, the ciphertext is then hashed using SHA-256:

(27) h=SHA256(C)

The hash value is digitally signed using the node’s private key via the Elliptic Curve Digital Signature Algorithm (ECDSA):

(28) $$\left(r,s\right)=ECDSA_{K_{priv}}\left(h\right)$$

On the receiving side, the backend/web application performs signature verification using the corresponding public key $Q_{pub}$ after MQTT reception:

(29) $$ECDSA\_Verify\left(h,r,s,Q_{pub}\right)\to \left\{0,1\right\}$$

Only packets that successfully pass backend-side verification are decrypted to recover the original payload and inserted into the database as authenticated edge-conditioned sensing measurements. This verification-first strategy establishes an end-to-end chain of trust from physical measurement acquisition to backend data storage while preventing unauthorized data injection and tampering.

### 2.5.5. Illustrative Example of Secure Data Packaging

To illustrate the proposed data-centric security mechanism, a representative monitoring record acquired at a sensor node is presented below in its logical (pre-serialization) form:

{

"node_id": 1,

"timestamp": 1775038530,

"crack_raw": 1234,

"temperature": {

"T1": 28.5,

"T2": 30.1

}

}

**Binary payload construction.**

To minimize transmission overhead, the sensing node serializes the measurements into a fixed-length packed binary payload without compiler padding. The resulting raw binary representation is:

[01 | 1234 | 28.5 | 30.1 | 1775038530]

where the fields are encoded as follows:
  - node_id: uint8_t (1 byte, value 0x01);
  - crack_raw: uint16_t (2 bytes, value 1234);
  - T1: float (4 bytes, value 28.5);
  - T2: float (4 bytes, value 30.1);
  - timestamp: uint32_t (4 bytes, Unix epoch).

The total raw payload size prior to encryption is therefore 15 bytes.

**AES encryption and padding.**

Since AES operates on fixed 16-byte blocks, a single PKCS#7 padding byte (0x01) is appended to the payload, yielding the final plaintext:

[01 | 1234 | 28.5 | 30.1 | 1775038530 | 01]

with a total size of 16 bytes. The plaintext is then encrypted using AES with a symmetric session key derived via ECDH:

$K_{\mathrm{AES}}=6A9F12C8E4D1930F7B5A6829\mathrm{ACF}10D44.$

The resulting ciphertext (a fixed ciphertext length of 16 bytes) is:

9FA23C810B6E725D419B21C8E21794A0

**Digital signature using ECDSA.**

To ensure payload integrity and source authenticity, the ciphertext is hashed using SHA-256 and digitally signed using ECDSA. The resulting signature consists of two 256-bit components:

r = 7F9A31D8C2B05A91F7A421B6C8D09E113A4FBC672D8E90AA4B5512CFF0987123

s = 2E3B49547D0A1C88A8B3F12D45EE9087C4F89123AB56CC781DFA2209BC4418F0

Each component occupies 32 bytes, resulting in a total signature size of 64 bytes.

**Final secure LoRa payload**

The final uplink packet transmitted by the sensor node contains only the encrypted ciphertext and the ECDSA signature:

[ ciphertext | r | s ]

The total transmitted payload size is therefore:

16bytes(ciphertext)+32bytes(r)+32bytes(s)=80bytes.

For clarity, an illustrative JSON-style representation of the secure payload forwarded by the gateway to the cloud is shown below.

{

"ciphertext": "9FA23C810B6E725D419B21C8E21794A0",

"signature": {

"r":"7F9A31D8C2B05A91F7A421B6C8D09E113A4FBC672D8E90AA4B5512CFF0987123",

"s":"2E3B49547D0A1C88A8B3F12D45EE9087C4F89123AB56CC781DFA2209BC4418F0"

}

}

This example demonstrates how compact edge-conditioned sensing measurements are transformed into an encrypted and authenticated payload, ensuring end-to-end data confidentiality, integrity, and authenticity while remaining compatible with LoRa-based low-power tunnel monitoring.

### 2.5.6. Backend Verification and Trust Enforcement

At the backend/web application layer, incoming packets are processed only if the ECDSA signature is successfully verified using the registered public key of the originating sensor node. Upon successful verification, AES decryption recovers the serialized payload, which is then parsed and stored in the cloud database. The gateway does not perform this verification/decryption step and does not possess plaintext access to monitoring measurements. This procedure enforces a strict zero-trust data admission policy and prevents unauthorized data injection.The backend-side secure uplink verification workflow is summarized in Algorithm 1.

**Algorithm 1:** Secure Uplink Verification at Backend/Web Application

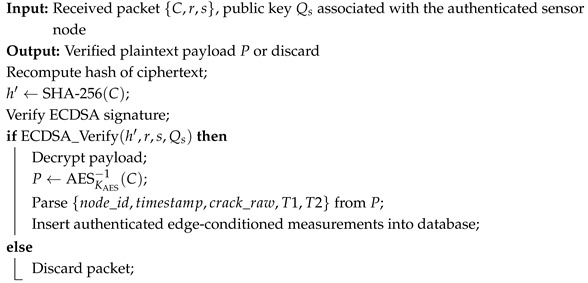



### 2.5.7. Distributed Trust and Secure Node Authentication

Because tunnel nodes may be physically accessible, the backend verifies both cryptographic validity and physical plausibility before accepting packets into the monitoring database. This keeps the gateway forwarding-only while rejecting tampered, replayed, or unauthorized data.

**Replay attack mitigation.**

Replay attack mitigation is achieved through timestamp consistency validation:

(30) $$\Delta t=t_{recv}-t_{msg}<T_{max}$$

where $t_{recv}$ denotes the backend reception time, $t_{msg}$ is the packet timestamp, and $T_{max}$ represents the maximum allowable temporal deviation. Packets violating this condition are discarded.

**Cyber–physical plausibility verification**

In addition to cryptographic verification, the backend/web application performs physical plausibility validation to reject abnormal structural measurements inconsistent with tunnel deformation behavior:

(31) $$|\Delta w_{t}-\Delta w_{t-1}|<\delta_{phys}$$

where $\delta_{phys}$ denotes the maximum physically admissible crack variation threshold.

The quantitative cybersecurity validation results of the proposed SHM framework are summarized in Table 4.

Security validation confirmed that modified packets, replay transmissions, and unauthorized node injection attempts were rejected in the implemented tests. The measured verification latencies remained small relative to the 60 s sensing interval used in the deployment, supporting trustworthy field-data admission without disrupting monitoring operation.

### 2.6. Embedded Firmware Implementation

The firmware is implemented in FreeRTOS to separate sensing, processing, security, communication, and storage into prioritized tasks. Sensor nodes handle local acquisition, edge processing, and secure packet generation; the gateway coordinates TDMA communication and forwards packets to the cloud backend.

#### 2.6.1. Embedded System Execution Overview

**Figure 11:**
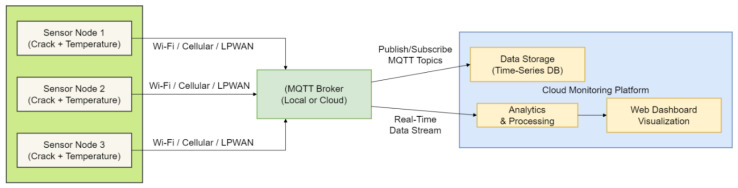
Operational architecture of the embedded system.

The FreeRTOS execution flow separates sensing, processing, security, communication, and local storage tasks so that monitoring remains robust under resource-constrained embedded operation. Figure 11 summarizes the embedded-system operational architecture implemented for resource-constrained tunnel monitoring.

#### 2.6.2. Sensor Node Firmware Architecture

Each sensor node operates as an autonomous FreeRTOS unit with queue-based task communication.

The sampling task performs periodic acquisition of crack displacement and temperature data. It is triggered by a FreeRTOS software timer and assigned the highest priority. The task interfaces directly with the sensor hardware and pushes raw measurements into an inter-task queue for downstream processing.

The processing task performs edge-level signal conditioning, including Kalman filtering and wavelet-based denoising. In addition, this task serializes processed measurements into compact payloads suitable for wireless transmission. Cryptographic preparation steps are scheduled only after signal validation and operate within a bounded execution window to avoid interference with sensing.

The communication task is responsible for constructing secure packets and transmitting them to the gateway using LoRa-based wireless links. This includes payload encryption and digital signature generation as described in Section 2.5.4. The task retrieves prepared payloads from queues and executes non-blocking transmission routines to prevent communication delays from affecting time-critical sensing tasks.

To improve robustness, a local storage task logs monitoring data to an SD card as an offline backup. This task operates at low priority and is event-driven. In cases of temporary communication failures, stored data can be retransmitted or synchronized once connectivity is restored.

Table 5 summarizes the FreeRTOS task scheduling adopted at each sensor node.

The sensor-node task schedule separates acquisition, processing, communication, and backup functions to keep time-critical sensing isolated from network and storage operations.

The sensor-node FreeRTOS structure provides implementation evidence for this separation of responsibilities across sensing, processing, secure communication, and local logging.

**Figure 12:**
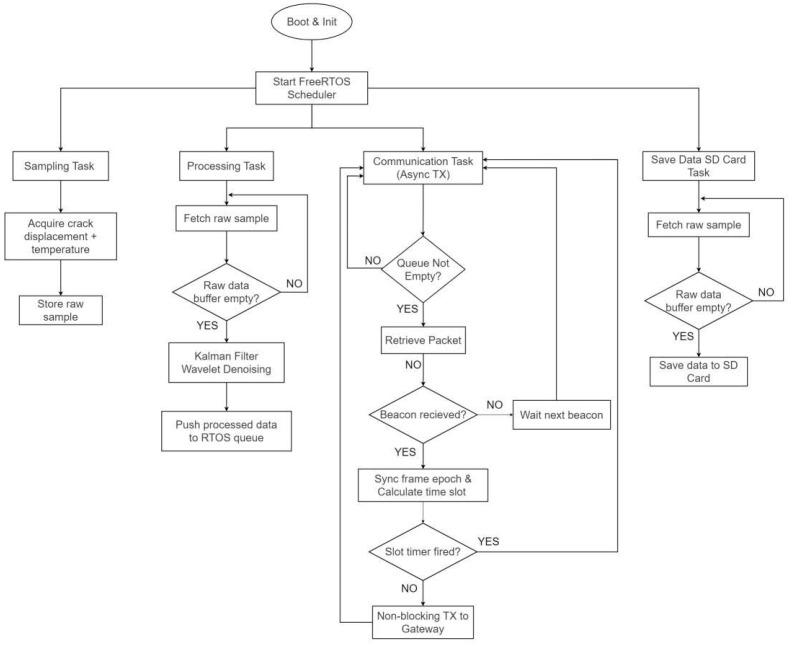
Sensor-node FreeRTOS task architecture.

The task-based FreeRTOS architecture enhances not only real-time operational reliability but also the long-term scalability and maintainability of the proposed SHM system. By separating sensing, signal processing, security, communication, and storage into independent tasks, the framework allows for future integration of advanced technologies such as AI-based anomaly detection, TinyML inference, adaptive filtering, or next-generation security mechanisms without major modifications to the existing system. This modular design improves software reusability, simplifies debugging and upgrades, and reduces the risk of system-wide instability during long-term deployment in evolving tunnel monitoring applications. Figure 12 presents the FreeRTOS task architecture used in each autonomous sensor node.

#### 2.6.3. Gateway Firmware Architecture

The gateway acts as the central TDMA coordination and packet-forwarding unit of the system. Compared to sensor nodes, the gateway handles higher data throughput and more complex real-time scheduling requirements, but it does not decrypt payloads, perform analytical processing, or access plaintext monitoring measurements.

The TDMA synchronization task periodically generates and broadcasts synchronization beacons to sensor nodes to maintain TDMA-based uplink scheduling. It executes with high priority to ensure global time alignment and collision-free uplink operation.

Triggered by radio interrupts, the LoRa reception task receives incoming wireless packets from sensor nodes. It extracts packet metadata and forwards the encrypted and signed payloads to a queue for backend communication. Immediate forwarding prevents packet loss under high node density while leaving cryptographic verification and decryption to the backend.

The MQTT publishing task publishes encrypted monitoring packets to the cloud backend using the MQTT protocol. Operating asynchronously and at lower priority, it ensures that cloud-side communication does not block time-critical radio or synchronization tasks.

Table 6 summarizes the FreeRTOS task scheduling implemented at the gateway.

**Figure 13:**
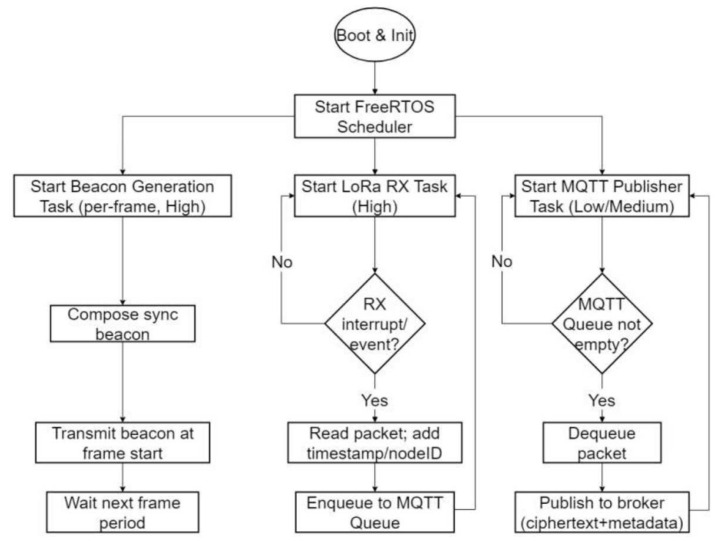
Gateway FreeRTOS task flow.

The gateway task schedule separates TDMA beaconing, LoRa reception, and MQTT publishing so that packet forwarding remains predictable during field operation. Figure 13 illustrates the gateway FreeRTOS flow for TDMA coordination, LoRa reception, and MQTT forwarding.

The gateway execution flow demonstrates beacon-based TDMA coordination, encrypted packet reception from sensor nodes, and MQTT-based forwarding to the cloud without payload decryption.

#### 2.6.4. Cryptographic Operations in the Firmware Execution Flow

From a firmware perspective, cryptographic operations are treated as bounded computational routines executed within designated tasks rather than as standalone protocol layers. On sensor nodes, encryption and digital signature generation are executed within the communication task after payload serialization. Signature verification and decryption are executed at the backend/web application layer after MQTT reception, not on the gateway.

The cryptographic stack implemented on each ESP32 sensor node includes: elliptic curve Diffie–Hellman for session key establishment, the elliptic curve digital signature algorithm for authentication, AES-128 for payload encryption, SHA-256 for hashing, and a hardware true random number generator for cryptographic entropy.

These operations are scheduled to ensure that cryptographic execution does not interfere with periodic sensing or radio reception, preserving real-time guarantees.

#### 2.6.5. Backend Packet Verification Routine

Incoming packets forwarded by the gateway are admitted to the database only if backend-side cryptographic verification is successful. The verification and decryption routine executed within the backend/web application is summarized in Algorithm 2.

**Algorithm 2:** Secure JSON Uplink Verification at Backend/Web Application

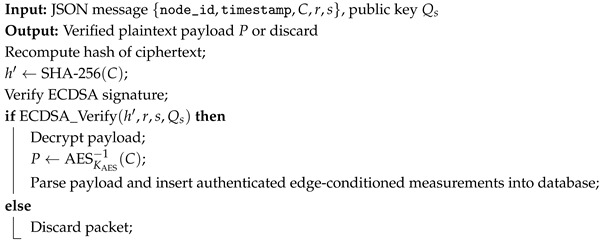



    By integrating FreeRTOS-based task scheduling with edge-level signal processing and secure data handling, the proposed embedded firmware achieves deterministic real-time performance while maintaining end-to-end data trust. The separation of sensing, processing, communication, and storage into independent tasks ensures scalability, robustness, and maintainability for long-term tunnel SHM.

### 2.7. Cloud and Data Management

To support long-term operation, real-time accessibility, and efficient post-processing of monitoring data, the proposed SHM–IoT system incorporates a cloud-based data management layer. This layer is responsible for ingesting, storing, organizing, and serving time-series data collected from distributed sensor nodes deployed inside the tunnel.

The cloud architecture follows a centralized and scalable design, in which all validated monitoring data forwarded from the MQTT communication layer are persistently stored in a database system. MongoDB is selected as the primary storage solution due to its native support for document-based data models, compatibility with JSON-format messages, and efficient handling of high-volume time-series workloads.

#### 2.7.1. Data Ingestion Pipeline

The data ingestion pipeline is designed to ensure reliable, low-latency transfer of monitoring data from the MQTT broker to the database backend. The pipeline is intentionally kept lightweight to avoid introducing computational overhead that could limit system scalability.

Upon receiving MQTT messages from the subscribed topics, the backend system performs the following operations:

1. Message subscription: the backend subscribes to the designated MQTT topics and listens for incoming JSON messages transmitted by the gateway.
2. Signature verification: the backend verifies the ECDSA signature over the received ciphertext using the registered public key of the originating sensor node.
3. Payload decryption: packets that pass authentication are decrypted using AES to recover the serialized edge-conditioned sensing measurements.
4. Payload parsing: the decrypted payload is parsed into an internal data structure containing sensor identifier, timestamp, crack measurement, and  environmental measurements suitable for database insertion.
5. Immediate storage: authenticated edge-conditioned sensing measurements are inserted into the database without computing trend-analysis outputs, displacement deltas, statistical derivatives, or predictive indicators.

By limiting ingestion to backend-side verification, decryption, parsing, and storage, the pipeline supports recurring data streams from multiple sensor nodes while maintaining predictable performance and low latency.

#### 2.7.2. Time-Series Database Schema

Given the timestamped nature of tunnel monitoring data, the database schema is designed to efficiently store authenticated edge-conditioned sensing measurements while preserving data integrity and analytical flexibility. In this study, raw ADC samples refer to unprocessed electrical acquisition signals before edge filtering; edge-conditioned sensing measurements refer to physically meaningful stabilized measurements after wavelet denoising and Kalman filtering; and derived analytical parameters refer to offline-computed quantities such as trends, statistical estimations, displacement variation metrics, and predictive indicators.

MongoDB is used in a document-oriented manner, where each measurement is stored as an independent document containing only authenticated edge-conditioned sensing values and essential metadata. A typical document structure is shown below.

{

"time": "2026-04-01T10:15:30Z",

"node": "S1",

"crack": 1234,

"T1": 28.5,

"T2": 30.1

}

Only authenticated edge-conditioned sensing measurements are stored in the database. Raw ADC streams, intermediate signal buffers, statistical derivatives, trend-analysis outputs, displacement deltas, and long-term inferred indicators are not stored. This design ensures that the database functions as a consistent and immutable source of truth for all trusted real-time monitoring measurements while keeping advanced structural analysis separate from real-time ingestion.

**Figure 14:**
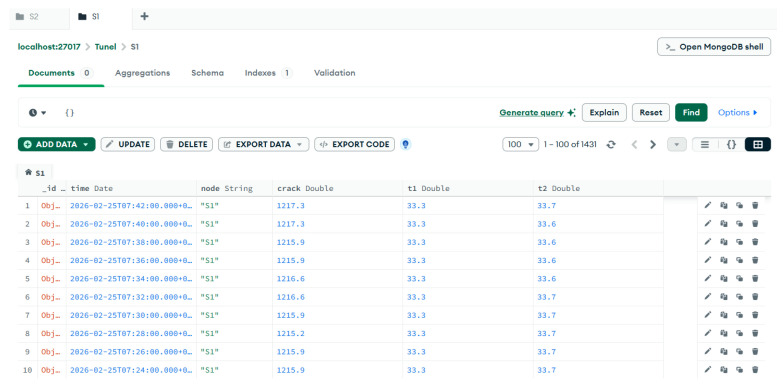
Time-series authenticated crack and temperature measurements for monitoring location S1.

**Figure 15:**
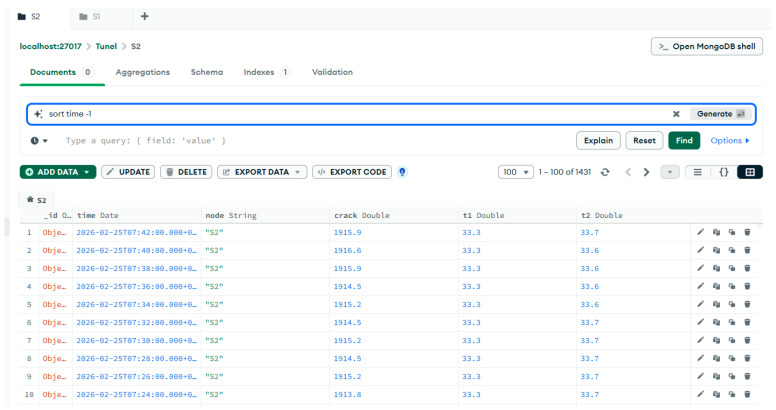
Time-series authenticated crack and temperature measurements for monitoring location S2.

Representative time-series data stored in the MongoDB database for monitoring locations S1 and S2 are shown in Figure 14 and Figure 15, respectively.

At S1 position:

At S2 position:

Figure 14 and Figure 15 show authenticated edge-conditioned crack and temperature measurements stored in MongoDB for monitoring locations S1 and S2, respectively.

#### 2.7.3. Real-Time Visualization and Offline Analysis

For real-time monitoring, the dashboard retrieves data directly from the database and visualizes authenticated edge-conditioned sensing measurements using calibration parameters. This approach enables immediate review of authenticated sensing records without requiring additional analytical processing at the database or gateway levels.

In contrast, higher-level analytical computations are intentionally deferred to offline analysis or data export stages. Operations such as mean estimation—using either global references or sliding windows—and displacement variation computation are performed only when required for reporting or long-term assessment. For example, crack displacement variation is calculated using Equation (25).

**Figure 16:**
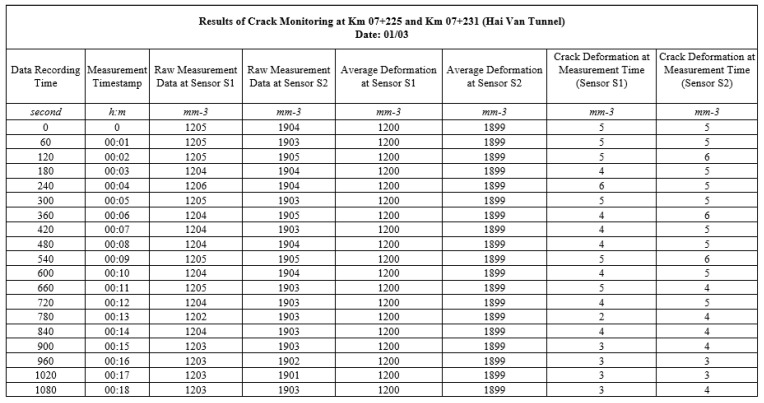
Example of offline crack displacement analysis after data export.

Offline analysis after data export converts stored measurements into engineering indicators such as mean crack width and displacement variation for reporting and structural assessment. Figure 16 provides an example of the offline crack-displacement analysis performed after data export.

For long-term data handling, authenticated edge-conditioned measurements are stored with timestamps and sensor identifiers, while derived indicators are computed offline. Timestamp, sensor, and compound (node_id, timestamp) indexes support efficient time-series retrieval in MongoDB.

It should be noted that performance metrics such as database insertion rate, query latency, storage efficiency, and scalability under increasing numbers of sensor nodes were not explicitly evaluated in this study. These aspects are important for large-scale deployments and are identified as directions for future work.

## 3. Field Deployment in Hai Van Tunnel

To validate the latest proposed IoT-based SHM framework, a recent field monitoring campaign was conducted in the Hai Van Tunnel, focusing on evaluating the current behaviour of representative cracks in Tunnel 1. These crack regions were initially believed to be associated with construction of the adjacent tunnel tube, where blasting and excavation activities may have influenced the existing lining. The tunnel segment containing these cracks therefore provides a practical testbed for validating the evolved IoT-based SHM system under operational underground conditions.

The emphasis of the present deployment is not on reconstructing the historical crack-formation process, but on assessing the recent performance of the updated monitoring architecture during a controlled 30-day operational campaign. This framing supports evaluation of the complete SHM workflow, including sensing stability, edge signal processing, secure telemetry, backend storage, and quantitative interpretation of crack behaviour.

### 3.1. Hardware Devices

The hardware integrates crack sensing, temperature measurement, ESP32 processing, LoRa communication, power regulation, and environmental protection for underground operation.

**Figure 17:**
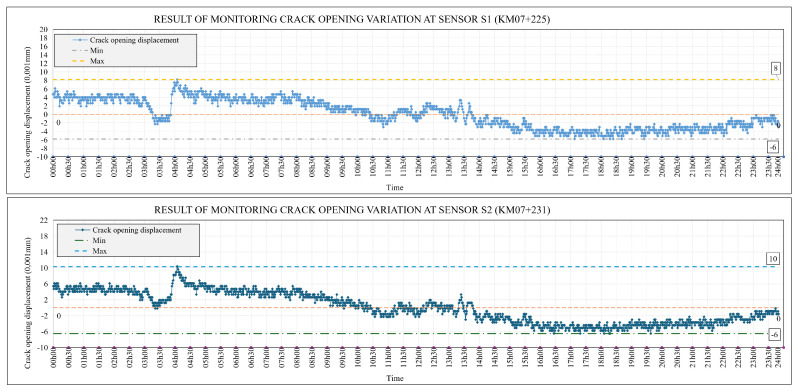
Campaign-scale crack-width time series showing baseline-centred behaviour at Km07 + 225 (S1) and Km07 + 231 (S2).

Crack width was measured using a TOTC-03 electronic displacement sensor (developed by the Transport Science and Technology Center, University of Transport and Communications, Vietnam). The TOTC-03 electronic displacement sensor utilizes a high-precision potentiometric principle to achieve a resolution of 0.001 mm. From an SHM perspective, the measurement quality is further characterized by a laboratory-validated repeatability of ±0.005 mm and an estimated measurement uncertainty of ±2 µm. To ensure longevity in the harsh tunnel environment (high humidity and dust), the sensors were housed in protective steel enclosures. The 30-day field data (Figure 17) confirmed the absence of significant long-term drift, supporting the sensor’s suitability for long-term underground monitoring applications, although full service-life verification requires extended field deployment. A temperature module with approximately 0.1°C accuracy was installed to support thermal-response interpretation.

An ESP32 performed analog acquisition, digitization, timestamping, buffering, and edge-level signal conditioning at fixed sampling intervals.

Wireless transmission used an SX1278 LoRa transceiver connected to the ESP32 through SPI.

A regulated AC–DC unit powered the system, and all components were placed in a protective enclosure with an integrated LoRa antenna. This configuration emphasizes field robustness and reproducible deployment rather than laboratory-only instrumentation.

The ‘low-cost’ nature of the architecture is demonstrated by a per-node material cost of approximately USD 80–150, which is roughly 15–20% of the cost of industrial-grade wired data acquisition systems or fiber-optic sensing solutions. This affordability, combined with the use of standard AC–DC power and protective enclosures, enables scalable deployment for recurring monitoring campaigns in developing infrastructure networks.

**Figure 18:**
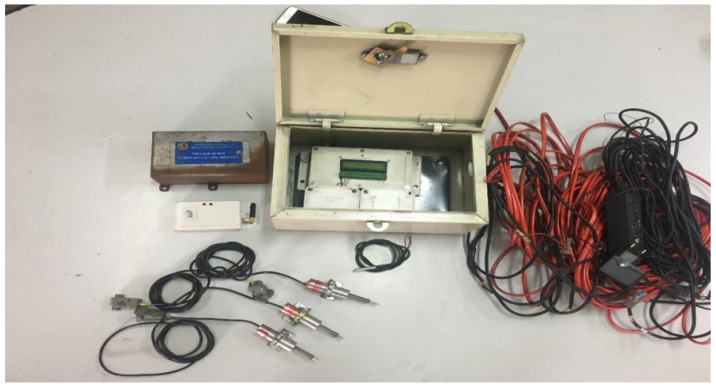
Field-ready hardware configuration integrating crack sensing, edge processing, LoRa communication, power regulation, and environmental protection.

Figure 18 shows the field-ready hardware configuration used for crack sensing, edge processing, wireless transmission, and environmental protection.

### 3.2. Monitoring Locations

The cracks monitored in this campaign were initially identified and localized through routine manual visual inspections and engineering surveys conducted following adjacent tunnel blasting activities. Two specific cracks, located at Km07 + 225 (S1) and Km07 + 231 (S2), were selected as representative “sentinel” monitoring points because they are situated within the tunnel segment most directly exposed to construction-induced vibration effects and were considered critical locations for long-term displacement observation.

**Figure 19:**
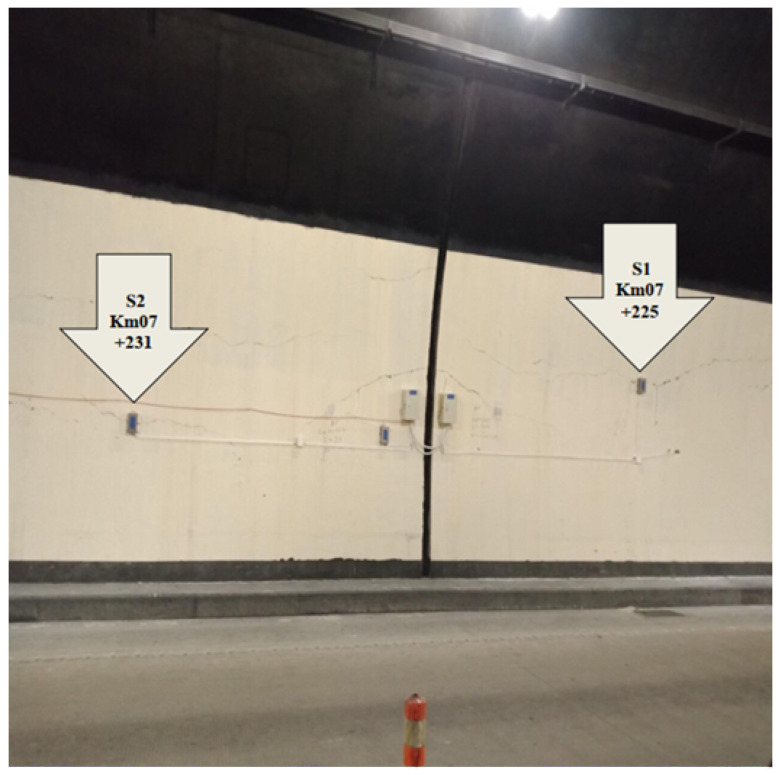
Operational monitoring locations selected at two representative tunnel-lining cracks, Km07 + 225 (S1) and Km07 + 231 (S2).

Figure 19 identifies the two representative tunnel-lining crack locations selected for operational monitoring.

It should be noted that the objective of the proposed SHM-IoT framework is continuous crack-width monitoring rather than automated damage detection, crack localization, or crack-origin analysis. The present study assumes that potential defects have already been identified through conventional inspection procedures prior to sensor deployment. Accordingly, the proposed system focuses on providing high-resolution, long-term measurements of pre-identified cracks to support condition assessment and maintenance decision-making after damage identification has been completed.

While this point-based sensing approach focuses on localized high-risk zones rather than complete tunnel coverage, it provides the micrometer-level measurement resolution required to detect subtle crack-width variations that may not be readily observable using wide-area inspection techniques.

Accordingly, the selected locations should be regarded as representative monitoring points within the most vibration-sensitive tunnel segment rather than indicators of the condition of the entire tunnel. Broader spatial coverage would require the deployment of additional sensor nodes across multiple tunnel sections.

### 3.3. Sensor Installation and Data Acquisition

Crack displacement and temperature were recorded automatically at 60 s intervals and transmitted through the IoT communication framework described in Section 2.4. The installation was designed to preserve sensor stability during the campaign while remaining practical for repeated field deployment.

**Figure 20:**
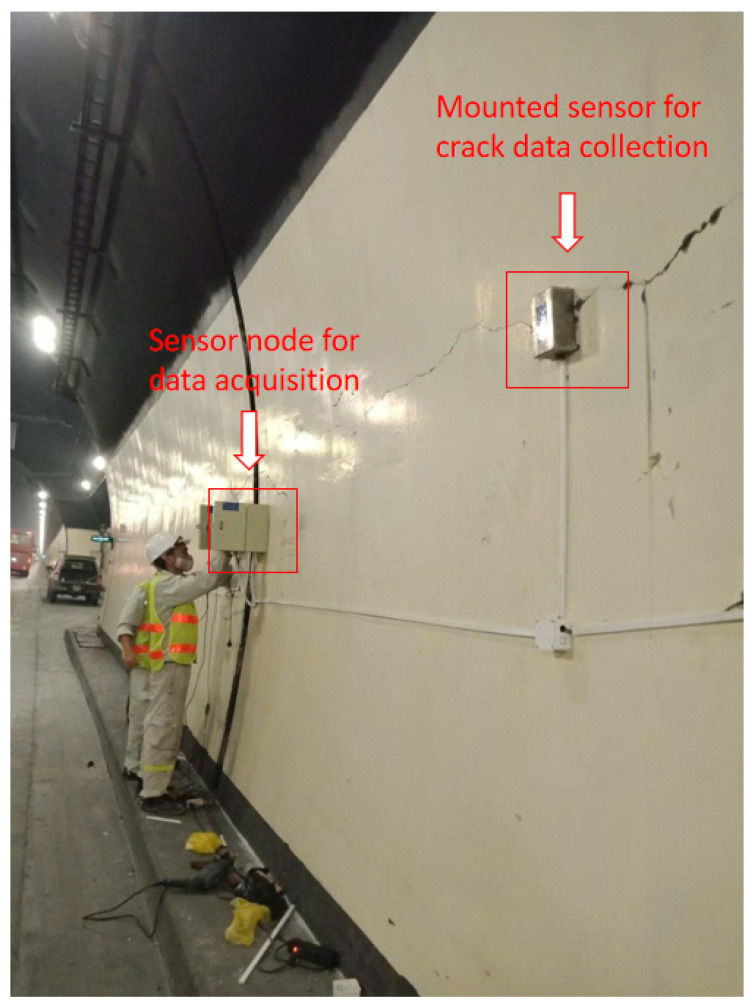
On-site installation of the electronic crack displacement sensor for campaign-based tunnel SHM.

Figure 20 shows the on-site installation arrangement of the electronic crack displacement sensor.

### 3.4. Functional Validation

**Cryptographic Overhead Measurement**

To evaluate the practicality of the proposed cryptographic framework on resource-constrained ESP32 sensor nodes, the computational and communication overhead introduced by security operations was experimentally measured. The evaluation focused on asymmetric signing operations, symmetric encryption, and their combined impact on system latency and payload size.

All measurements were conducted on ESP32 nodes operating at 240 MHz under the FreeRTOS-based firmware described in Section 2.6. The cryptographic operations were executed as part of the communication task to avoid interference with time-critical sensing processes.

The measured overhead remains small relative to the sampling cycle, supporting secure telemetry without compromising operational sensing continuity.

Table 7 reports the measured cryptographic overhead and secure-payload impact on the ESP32 sensor nodes.

The results indicate that ECDSA signing constitutes the dominant source of computational overhead, while AES encryption introduces only negligible latency.

The cryptographic overhead also results in an increased payload size. As described in Section 2.5.5, the final secure LoRa payload is 80 bytes, including encrypted data and ECDSA signature components. This payload size is consistently used in the communication analysis presented in Table 3.

**Functional Validation with Experimental Data**

Functional validation was performed using real crack-width and temperature data collected during the recent Hai Van Tunnel deployment. Measurements were transmitted with and without AES/ECDSA protection, and backend verification followed Algorithm 2.

Cybersecurity validation was conducted using controlled packet-injection experiments designed to emulate common threats in distributed SHM-IoT systems. Four attack categories were considered: payload tampering, replay attacks, unauthorized-node transmission, and malformed ciphertext generation. For each category, 500 independent malicious packets were generated and transmitted to the backend validation framework, resulting in a total of 2000 cybersecurity test cases. The detection rates reported in Table 4 correspond to these experimental datasets and quantify the ability of the proposed AES/ECDSA-based framework to identify and reject unauthorized or modified monitoring messages. The results demonstrate that:

- All valid packets from authorized sensor nodes were successfully authenticated;
- No legitimate packets were rejected during verification;
- Decrypted payloads exactly matched the original sensing measurements;
- Injected malicious packets in the four attack categories were rejected by the backend validation logic.

These results confirm the correctness and robustness of the proposed cryptographic pipeline under real operating conditions.

**Latency and Real-Time Performance Analysis**

For the selected LoRa configuration (SF7, bandwidth 125 kHz), the secure 80-byte payload results in a Time-on-Air of approximately 153.9 ms, as shown in Table 3. This value represents the dominant component of communication latency.

The total end-to-end transmission latency, including cryptographic processing, can be approximated as follows:

(32) $$T_{\mathrm{latency}}\approx T_{\mathrm{ToA}}+T_{\mathrm{crypto}}=153.9\;\mathrm{ms}+22.5\;\mathrm{ms}\approx 176.4\;\mathrm{ms}.$$

This latency remains significantly smaller than the adopted sensing interval of 60 s, indicating that secure transmission does not constrain periodic operational monitoring.

**Computational Efficiency**

The computational load introduced by cryptographic operations is evaluated in terms of CPU duty cycle:

(33) $$\mathrm{CPU}\;\mathrm{duty}\;\mathrm{cycle}=\frac{T_{\mathrm{crypto}}}{T_{\mathrm{sampling}}}=\frac{22.5\;\mathrm{ms}}{60\;s}\approx 0.0375\%.$$

This result confirms that the computational overhead of AES encryption and ECDSA signing is negligible relative to the system operation cycle, which is essential for resource-constrained SHM nodes intended for repeated field use.

**Discussion**

Overall, the experimental results indicate that:

- Secure communication increases payload size but remains compatible with LoRa transmission constraints.
- The combined communication and cryptographic latency remains below 200 ms, which is negligible compared to the sensing interval.
- The computational overhead is minimal on ESP32 platforms and does not affect real-time sensing performance.

These findings demonstrate that the proposed secure edge-IoT SHM framework achieves a practical balance between security, communication efficiency, and real-time operation under resource-constrained system conditions.

## 4. Monitoring Results and Discussion

### 4.1. Operational Sensing Stability

The recent monitoring campaign first evaluated whether the deployed system could provide stable and interpretable crack-width measurements under tunnel operating conditions. At the beginning of the observation period, the crack widths were approximately 0.50 mm at Km07 + 225 (S1) and 0.65 mm at Km07 + 231 (S2). Over the 30-day campaign, both time series remained centred around these baseline values, and short-lived displacement peaks were followed by recovery rather than cumulative widening.

This behaviour indicates that, within the 30-day observation window, the monitoring data provide engineering evidence of reversible crack response rather than a progressive widening trend. The campaign-scale time histories in Figure 17 show that both monitored cracks oscillated around their respective mean values without sustained drift.

### 4.2. Thermo-Mechanical Response

The dominant interpretation challenge in operational tunnel SHM is distinguishing structural change from environmentally induced fluctuation. In the present campaign, crack-displacement variations followed the ambient temperature trend, indicating that the measured micrometer-scale changes were mainly thermo-mechanical responses of the tunnel lining. This relationship is important because it reduces the likelihood that short-lived peaks are misinterpreted as damage progression.

**Figure 21:**
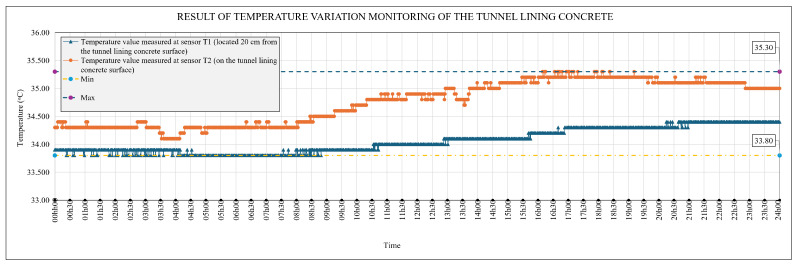
Thermo-mechanical coupling between crack-displacement variation and ambient temperature during the recent monitoring campaign.

To further quantify the observed thermo-mechanical behavior, a simple correlation and linear regression analysis was performed between the measured crack-width variation and the tunnel-lining temperature records. The results supported a consistent positive association between temperature fluctuation and crack response during the monitoring period, in agreement with the temporal consistency shown in Figure 21. Although additional environmental and structural factors may also influence crack behavior, the assessment indicates that temperature was the dominant observable factor governing the measured reversible crack-width variations in this campaign. Therefore, the observed fluctuations are interpreted primarily as thermo-mechanical responses of the tunnel lining rather than evidence of progressive crack propagation or structural deterioration.

### 4.3. Structural Interpretation of Crack Behaviour

The campaign-scale crack-response interpretation was therefore based on three engineering indicators: bounded displacement amplitude, recovery after local peaks, and absence of sustained accumulation. For S1, the maximum crack expansion ranged approximately from 5 µm to 23 µm, with most values remaining within 8-19 µm. The largest expansion occurred around 18 March and decreased immediately afterwards, indicating a transient response rather than a persistent displacement shift. Crack contraction at S1 ranged from approximately −5 µm to −9 µm, with typical values concentrated around −6 to −7 µm. The coexistence of limited expansion and contraction indicates that the crack returned close to its initial condition after each deformation cycle.

**Figure 22:**
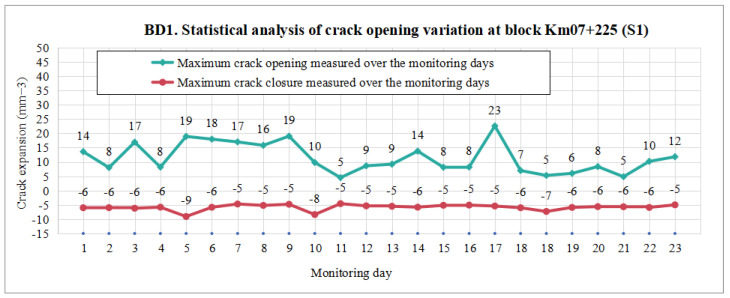
Daily expansion–contraction envelope for S1, showing bounded reversible crack response without cumulative widening.

Figure 22 presents the daily expansion–contraction envelope for S1.

For S2, the maximum crack expansion ranged approximately from 5 µm to 31 µm, with most values falling within 8–25 µm. Two larger transient peaks were observed around 26 February and 17–18 March, reaching approximately 31 µm and 29 µm, respectively. These peaks were not sustained in subsequent measurements and did not form a monotonic trend. Crack contraction at S2 primarily varied between −4 µm and −10 µm, with common values around −6 to −7 µm. Although S2 exhibited slightly larger expansion amplitudes than S1, its response remained bounded and reversible.

Taken together, the S1 and S2 responses indicate that, within the 30-day observation campaign, the monitored cracks did not show a cumulative widening trend. The measured variations were mainly within the micrometer range, were reversible, and showed a clear relationship with ambient temperature, suggesting thermo-mechanical operational response rather than progressive structural degradation.

**Figure 23:**
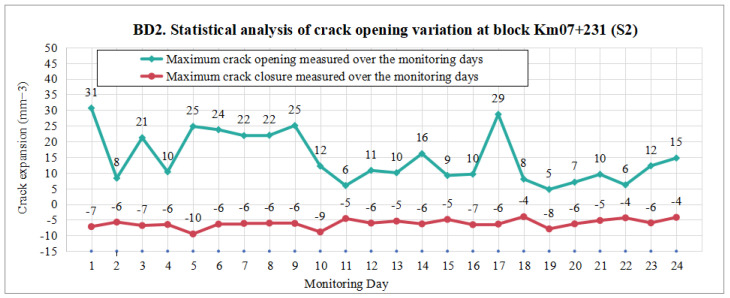
Daily expansion–contraction envelope for S2, showing transient peaks but no sustained displacement accumulation.

Figure 23 presents the corresponding daily expansion–contraction envelope for S2.

To make this interpretation explicit, the campaign-scale linear widening rate was estimated from the difference between the final and initial crack widths:

(34) $$S_{\mathrm{trend}}=\frac{D_{\mathrm{final}}-D_{\mathrm{initial}}}{\Delta t}.$$

For S1, $D_{\mathrm{initial}}\approx 0.50$ mm and $D_{\mathrm{final}}\approx 0.50$ mm over Δt=30 days, giving $S_{\mathrm{trend}}\approx 0$ mm/day. For S2, $D_{\mathrm{initial}}\approx 0.65$ mm and $D_{\mathrm{final}}\approx 0.65$ mm over the same period, also giving $S_{\mathrm{trend}}\approx 0$ mm/day. These near-zero campaign-scale slopes support the absence of progressive crack widening at the two monitored locations. A regression-based coefficient such as $R^{2}$ was not used as a stability criterion here because the objective was not to fit a monotonic growth model to the thermally reversible fluctuations, but to evaluate whether a residual widening trend was present over the observation window.

In this study, structural stability is defined operationally rather than as the complete absence of crack-width variation. A monitored crack is considered stable during the campaign when: (i) no progressive long-term widening trend is observed; (ii) displacement fluctuations remain bounded within a repeatable thermo-mechanical response envelope; (iii) no cumulative residual deformation develops over the observation period; and (iv) measured crack-width variations remain below the proposed maintenance-alert threshold. This criterion-based interpretation is supported by the following evidence:

- The campaign-scale linear widening rate was approximately zero for both monitored cracks, and no monotonic or cumulative increase in crack width was detected.
- Displacement variations remained bounded within the micrometer scale and below the proposed maintenance-alert threshold.
- Local peaks were followed by recovery rather than persistent widening, indicating no residual accumulation during the campaign.
- Crack variations were coupled with environmental temperature changes, indicating a repeatable thermo-mechanical response envelope.

Based on these operational criteria, both monitored cracks exhibited stable behaviour during the 30-day monitoring campaign. This conclusion is limited to the instrumented S1 and S2 locations and should not be interpreted as a tunnel-wide stability assessment.

### 4.4. Implications for Tunnel SHM

The field campaign demonstrates that the proposed framework provides more than visualization of crack time histories; it converts field measurements into engineering evidence for operational decision support. Compared with visual inspection alone, the framework enables:

- High-resolution crack-displacement measurement with micrometer-scale precision;
- Repeatable acquisition of crack behaviour data during periodic operational campaigns;
- Interpretation of environmental influences on crack response;
- Quantitative assessment of whether observed variations are reversible or progressive;
- Secure telemetry and backend traceability for trustworthy SHM records.

Although the present field campaign focuses on crack-width monitoring, the proposed SHM-IoT architecture is not limited to a single sensing modality. The modular sensor-node design, edge-processing workflow, LoRa/MQTT communication layer, and cloud-based data architecture can be extended to accommodate additional tunnel-health indicators, including strain, structural convergence, vibration, temperature, humidity, and groundwater-related measurements. These additional sensing channels can share the same communication, timestamping, edge-processing, and cybersecurity framework, providing a foundation for future multi-sensor tunnel SHM deployments while preserving the resource-constrained edge architecture validated in this study.

The engineering value of the monitoring results lies in establishing an evidence-based action plan. Based on the measured reversible thermal peaks (typically <30 µm), an alarm threshold of >50 µm is proposed to trigger an immediate onsite structural integrity review. If the system detects a monotonic increasing trend that deviates from the established thermo-mechanical response envelope, it provides a quantitative basis for operators to initiate mitigation strategies, such as localized lining reinforcement or temporary traffic restrictions. While the proposed framework provides actionable alarm thresholds and condition-assessment support, the available 30-day monitoring duration is insufficient for reliable residual-life estimation, which remains a topic for future long-term studies. The campaign evidence is summarized in Table 8. Within the 30-day observation period, the two monitored cracks showed no cumulative widening trend, and the dominant measured variations were associated with temperature-induced expansion and contraction rather than sustained structural deterioration.

### 4.5. Comparison with Alternative Tunnel Monitoring Technologies

The proposed system is not intended to replace all tunnel inspection technologies. Vision-based systems, FBG sensing networks, and geodetic instruments each offer specific advantages depending on the monitoring objective [15,16,37,38]. Instead, the present framework is positioned as a low-cost, high-resolution, and trustworthy edge-IoT solution for continuous monitoring of pre-identified cracks. Table 9 summarizes the relative advantages and limitations of representative alternatives for tunnel crack monitoring.

Although modern CCD/CMOS camera systems are relatively inexpensive at the device level and can monitor larger tunnel areas, a robust vision-based tunnel monitoring installation typically also requires controlled illumination, stable mounting, lens protection and cleaning, high-bandwidth data transfer, image storage, and crack-segmentation or tracking algorithms [15,16,17]. These requirements are particularly important in underground environments where lighting variation, dust, humidity, lens contamination, and traffic occlusion can degrade image quality. For this reason, vision-based monitoring is well suited to broad visual inspection and traffic-related observation, whereas the proposed sensor-based system is better suited to direct high-resolution time-series measurement of selected cracks that have already been identified by inspection.

The low-cost claim should therefore be interpreted at the monitoring-node and deployment-workflow levels rather than as an absence of hardware. Each node still requires a displacement sensor, ESP32 controller, LoRa transceiver, power conversion, cabling, and a protective enclosure; however, these components are low-cost, commercially accessible, and do not require optical interrogators, survey-grade instruments, high-bandwidth camera infrastructure, or continuous manual survey operation [16,37,38]. In the present prototype, the estimated material cost is approximately USD 80–150 per sensing node, supporting repeated campaign-based deployment in resource-constrained tunnel management contexts.

### 4.6. Limitations and Future Work

The present field campaign was intentionally designed as a pilot deployment involving two instrumented cracks over a 30-day monitoring period. Therefore, the results primarily demonstrate the technical feasibility, reliability, and operational practicality of the proposed SHM-IoT framework under real tunnel conditions, rather than providing a comprehensive validation of large-scale deployment.

The communication assessment in this study primarily focused on deterministic LoRa Time-on-Air analysis and end-to-end operational reliability. During the 30-day field deployment, scheduled monitoring records were successfully received and stored without observable communication outages, indicating stable communication performance under the tested tunnel conditions. However, detailed characterization of packet delivery ratio (PDR), RSSI, LoRa SNR, and radio-propagation behaviour under different tunnel geometries was beyond the scope of the present deployment and remains an important direction for future work.

Additional long-term multi-node deployments are also required to fully evaluate network scalability and tunnel-wide structural representativeness. Future work will therefore focus on extended monitoring campaigns, larger sensor networks, integration of complementary sensing modalities such as strain, convergence, vibration, humidity, and groundwater indicators, physics-informed analysis, and edge-AI-assisted anomaly detection.

## 5. Conclusions

This study validated an evolved low-cost IoT-based SHM framework through a recent 30-day operational monitoring campaign in the Hai Van Tunnel. The field evidence demonstrates that the framework can support periodic tunnel SHM by combining stable crack sensing, edge-level signal conditioning, reliable LoRa/MQTT telemetry, lightweight security, and cloud-based data management under challenging underground conditions. Beyond component integration, the main contribution is the validation of a deployable workflow for producing trustworthy quantitative evidence on crack behaviour during operational infrastructure assessment.

The monitored cracks were initially associated with adjacent tunnel construction activities, but the present work focuses on their recent behaviour and on validation of the evolved SHM workflow. Observed crack-width variations remained small, reversible, and correlated with temperature, with no evidence of sustained displacement accumulation during the observation period. These findings support the suitability of campaign-based SHM for continued operational assessment of this tunnel segment. Although the present study focuses on tunnel crack monitoring, the proposed cyber–physical edge-SHM architecture is not limited to underground infrastructure. The combination of edge processing, secure wireless communication, and cloud-based data management can be extended to other SHM applications, including bridges, retaining structures, underground utility networks, and geotechnical monitoring systems requiring reliable low-power sensing in distributed environments. However, the deployment duration and number of monitored cracks were limited. Future work should therefore focus on longer repeated multi-node deployments, temperature-compensated structural interpretation, and predictive analytics for tunnel maintenance decision support.

## Figures and Tables

**Table 1:** Comparison of the proposed framework with representative recent SHM and IoT monitoring studies.

Study	Tunnel SHM	Edge Processing	LoRa	Security Mechanism	Real Tunnel Deployment
Tunnel SHM overview [13]	Yes	Partial	Partial	Limited	Yes
Tunnel FBG monitoring [14]	Yes	No	No	No	Yes
Low-cost IoT SHM [19]	No	Partial	No	Limited	No
LoRa-based SHM network [25]	No	No	Yes	No	No
ECC-based edge-IoT security [27,28]	No	Yes	No	ECC authentication	No
Proposed framework	Yes	Yes	Yes	AES + ECDSA	Yes

**Table 2:** Quantitative evaluation of signal-processing effectiveness.

Metric	Raw	Wavelet	Wavelet + Kalman	Improvement
SNR (dB)	8.4	14.9	19.6	+133.3%
RMSE (mm)	0.112	0.067	0.041	−63.4%
Std (mm)	0.086	0.049	0.031	−64.0%
HF noise energy	100%	46.8%	21.3%	−78.7%
PPR	1.000	0.972	0.958	−4.2%
Correlation	1.000	0.986	0.973	−2.7%

**Table 3:** LoRa ToA and TDMA slot length for different spreading factors.

SF	BW (kHz)	Payload (B)	ToA (ms)	Proposed Slot Length (ms)	Max Nodes per Frame (1 s)
7	125	80	153.9	180	5
8	125	80	348.7	420	2
9	125	80	778.6	950	1
10	125	80	1722.5	2100	0

**Table 4:** Quantitative cybersecurity validation results of the proposed SHM framework.

Attack Scenario	Detection Mechanism	Detection Rate	Avg. Verification Latency
Payload tampering	ECDSA verification	100%	18.7 ms
Replay packet	Timestamp validation	100%	3.2 ms
Unauthorized node injection	Public key authentication	100%	21.4 ms
Invalid ciphertext	AES integrity verification	99.8%	4.6 ms

**Table 5:** FreeRTOS Task Scheduling at Sensor Node.

Task	Function	Priority	Trigger	Description
Sampling Task	Acquire crack displacement and temperature data	High	Timer (e.g., 60 s)	Deterministic periodic sensing with minimal jitter
Processing Task	Kalman filtering and wavelet-based denoising	Medium	Event-driven	Bounded execution time to ensure real-time performance
Communication Task	Transmit monitoring data to gateway	Low	Queue-based	Non-blocking wireless data transmission
Save to SD Card Task	Store data locally for offline backup	Low	Queue/event-driven	Ensures data persistence during communication loss or desynchronization

**Table 6:** FreeRTOS task scheduling at gateway.

Task	Function	Priority	Trigger	Description
TDMA Beacon Generation Task	Generate and broadcast TDMA synchronization beacon to all sensor nodes	High	Periodic (per TDMA frame)	Ensures global time alignment and collision-free uplink scheduling
LoRa RX Task	Receive LoRa packets from sensor nodes and extract timestamp and node ID	High	Radio interrupt/RX event	Deterministic reception of uplink packets without blocking; forwards data to MQTT queue
MQTT Publisher Task	Publish encrypted payload and metadata to cloud backend	Low/Medium	Queue-based (MQTT queue not empty)	Handles backend communication asynchronously without affecting real-time radio tasks

**Table 7:** Measured cryptographic overhead and payload impact on ESP32 sensor nodes.

Metric	Value
AES encryption time per packet	≈0.5 ms
ECDSA signing time per packet	≈22 ms
Total cryptographic processing time ($T_{\mathrm{crypto}}$)	≈22.5 ms
Raw payload size (pre-security)	15 bytes
Secure payload size (AES + ECDSA)	80 bytes
Payload size increase	+65 bytes (total)
Additional energy per cycle	Negligible (AC-powered)

**Table 8:** Engineering evidence from the recent Hai Van Tunnel monitoring campaign.

Parameter	Observed Value	Engineering Interpretation
Monitoring location	Hai Van Tunnel (Km07 + 225, Km07 + 231)	Representative tunnel lining cracks
Number of monitored cracks	2	Independent S1 and S2 response validation
Crack displacement measurement resolution	0.001 mm	Sensitivity sufficient for micro-deformation detection
Temperature measurement accuracy	0.1 °C	Supports thermo-mechanical correlation analysis
Sampling interval	60 s	Operational time-series monitoring
Monitoring duration	30 days	Recent campaign-scale validation
Approximate total samples	∼43,200	Sufficient for statistical and trend interpretation
Initial crack width (Km07 + 225)	0.50 mm	Baseline condition for S1
Final crack width (Km07 + 225)	0.50 mm	No observable cumulative widening
Initial crack width (Km07 + 231)	0.65 mm	Baseline condition for S2
Final crack width (Km07 + 231)	0.65 mm	No observable cumulative widening
Observed crack variation	Small cyclic fluctuation (µm scale)	Bounded reversible response
Dominant influencing factor	Temperature variation	Thermo-mechanical expansion and contraction
30-day crack-response interpretation	No cumulative widening observed	Micrometer-scale reversible variations associated with temperature

**Table 9:** Comparison of representative tunnel crack monitoring technologies. Cost levels are qualitative and refer to typical deployment complexity and equipment requirements rather than vendor-specific quotations.

Criterion	Vision-Based Monitoring [15,16,17]	FBG Sensing [14,37]	Geodetic Survey/Total Station [38]	Proposed SHM-IoT System
Monitoring coverage	High; wide-area visual coverage	Localized along instrumented fibers	Localized measurement points	Localized at instrumented cracks
Crack-width resolution	Moderate; depends on optics, calibration, distance, and image quality	High	Moderate to high, depending on setup	High; 0.001 mm sensor resolution
Sensitivity to lighting	High	None	Low	None
Sensitivity to dust/humidity	High; lens contamination and image degradation are possible	Low, but installation protection is needed	Low to moderate	Low; protected sensor enclosure
Traffic/occlusion effects	Possible occlusion by vehicles and tunnel equipment	None after installation	Possible line-of-sight interruption	None at the sensing point
Continuous monitoring	Yes, with camera network and image processing	Yes	Usually periodic rather than continuous	Yes, with 60 s sampling in this campaign
Wireless telemetry	Possible, but often bandwidth-intensive	Limited; typically requires interrogator infrastructure	No or limited	Yes; LoRa/MQTT telemetry
Cybersecurity support	Possible, but rarely addressed in crack-monitoring studies	Rarely addressed at sensing layer	Not typical	Integrated AES/ECDSA-based data protection
Typical deployment cost	Moderate to high because of cameras, lighting, mounting, storage, and image-processing infrastructure	Very high because of optical fibers, interrogators, and specialist installation	Moderate to high because of survey-grade instruments and repeated manual operation	Low; approximately 80–150 USD per sensing node in this prototype
Best suited use	Wide-area inspection, visual documentation, traffic observation	High-precision distributed strain/temperature monitoring	Periodic geometric verification	Continuous high-resolution monitoring of known cracks

## Data Availability

The data presented in this study are available on request from the corresponding authors.

## Abbreviations

The following abbreviations are used in this manuscript:

AES	Advanced Encryption Standard
API	Application Programming Interface
ECC	Elliptic Curve Cryptography
ECDH	Elliptic Curve Diffie–Hellman
ECDSA	Elliptic Curve Digital Signature Algorithm
ESP32	Espressif 32-bit microcontroller platform
FBG	Fiber Bragg Grating
FreeRTOS	Free Real-Time Operating System
IoT	Internet of Things
JSON	JavaScript Object Notation
LoRa	Long Range
LPWAN	Low-Power Wide-Area Network
MQTT	Message Queuing Telemetry Transport
PPR	Peak Preservation Ratio
RMSE	Root Mean Square Error
SD	Secure Digital
SHM	Structural Health Monitoring
SNR	Signal-to-Noise Ratio
TDMA	Time Division Multiple Access
WSN	Wireless Sensor Network
